# The Psychological Experience of Grandparents: Proposal of a Qualitative Clinical Assessment Tool in Pediatric Palliative Care

**DOI:** 10.3390/clinpract14010010

**Published:** 2024-01-04

**Authors:** Alexandra Jóni Nogueira, Maria Teresa Ribeiro

**Affiliations:** CICPSI, Faculty of Psychology, University of Lisbon, Alameda da Universidade, 1649-013 Lisbon, Portugal; mteresaribeiro@psicologia.ulisboa.pt

**Keywords:** pediatric palliative care, life-limiting conditions, grandparents, family, psychological assessment, qualitative methods

## Abstract

In Portugal, there are over 7800 children with life-limiting conditions. The context of pediatric palliative care represents a complex and distressing experience for families. Compared to parental caregivers and healthy siblings, grandparents are underexplored in the literature and clinical practice. The aim of the present study is to propose a psychological experience assessment tool of grandparents in this context. It consists of a sociodemographic and clinical data sheet and a semi-structured interview based on sharing a testimony with other grandparents. On the basis of the latter, 10 dimensions were explored through the grandparents’ own perspective: representation of the illness; representation of the sick grandchild; changes in routine and life; family impact; grandparents’ contributions to the family system; social support and coping strategies; emotional impact; triple concern; needs identification; and post-traumatic growth. The tool can be used in person or remotely and may be combined with other instruments. Its application enables a personalized identification of needs and challenges for each family, promoting the adjustment of the clinical intervention to their wellbeing and resilience from an eco-systemic perspective. The clinical tool is presented in detail and its importance in the context of research and systemic intervention is discussed.

## 1. Introduction

Palliative care is a healthcare approach that aims to improve the quality of life of people diagnosed with an incurable and/or severe illness with a limited prognosis [1]. Palliative care was established as a healthcare domain in Portugal by the Ministry of Health, through the National Palliative Care Program [2], resulting in a legislative response to people with identified palliative needs.

While this service is extended to everyone, regardless of their age, when this specific care is applied to newborns, children and adolescents with a complex chronic disease, whether life-limiting or life-threatening, it is referred to as pediatric palliative care (PPC) [3]. Recognized by the World Health Organization since 1998, its main goal is to promote the psychological, physical and spiritual wellbeing of the child and youth and their entire family [4]. Therefore, multidisciplinary healthcare teams should consider the experiences of parental caregivers, but also those of the healthy siblings and grandparents, focusing on their needs and other features, up to and beyond the death of the child or youth in the context of bereavement [5].

In Portugal, the provision of PPC has seen rapid development, with the country positioned at level 4 on a 5-level scale since 2018 in view of its ample offer of palliative care for children, with training and targeted plans for service development and integration in the health services. As it is currently a developing area, a broader provision of PPC has also been seen on a worldwide scale, with countries such as Australia, the United Kingdom and the United States of America positioned at level 5. The difference between these countries and Portugal is their fully integrated approach to health services, as well as national policies supporting children’s palliative care [6]. According to data from 2018, it is estimated that there are approximately 7828 children/youths with identified palliative needs [7] resulting from the diagnosis of one of the four types of complex chronic diseases referred to in the literature [8].

### 1.1. The Family

The literature has mostly focused on the study of psychological adjustment in the context of illness in the parental system, due to the profound and mostly negative impact that serious illnesses in children/youths may have on their psychological wellbeing and quality of life. Indeed, parents play a central role as the primary caregivers, legal guardians and advocates of their medically complex child [9].

However, and from a systemic perspective, it is essential to look at the family as a whole and at each of its elements, as well as at the relationships they build with each other [10]. National and international studies have commented on the significant impact that a life-limiting condition has on the whole family system, contributing to changes in its relational dynamics, communication processes and structure [11]. Feudtner and colleagues [12] also concluded that, in families of children who were born substantially preterm or with critical congenital heart conditions, or who developed cancer or had progressive neurological conditions, the parents and siblings were 55% to 70% more likely to resort to healthcare and to receive diagnoses and prescriptions when compared to families with healthy children.

Thus, it may be said that, in addition to the caregivers, healthy siblings and grandparents also represent an important presence and role in the management of family dynamics [13,14]. Hence, they occupy a prominent position that deserves to be explored within the scope of their psychological experience of the illness, i.e., as regards the set of emotions, thoughts and behaviors stemming from and/or experienced within the context of an illness.

An in-depth understanding of their psychological experience promotes a more accurate identification of their singularities, concrete needs and risks and protective factors in their process of adaptation to the illness. This approach fosters health professionals’ increased knowledge about these family members, giving them the opportunity to align their intervention strategies and goals according to the real challenges and vulnerabilities of this population [15,16].

This perspective is also consistent with the definition of national strategic guidelines for Palliative Care in 2021–2022, namely, in terms of centering care on the person, (re)integrating the family and strengthening the social network. This goal is achieved through the identification of family needs, the promotion of their adaptation to the illness and the preservation of autonomy and psychosocial support [17].

### 1.2. Grandparents

In this context, grandparents are a key source of support for the family, although there are few studies that specifically focus on the experience of this family subsystem and are from the grandparents’ own perspective. Some authors [18] reinforce the importance of the grandparents’ role in PPC contexts, recognizing their constant presence for their sick grandchildren but also for their own children and healthy grandchildren, culminating in a triple concern [16,19]. Indeed, their participation in the family’s life cycle has the potential to improve the care and attention provided to both generations of offspring [20].

Wakefield and colleagues [21] concluded that, in families of children with cancer, grandparents may represent comfort and safety for the other elements of the system. However, these authors also stressed the importance of exploring their own psychological state, finding that grandparents appear to manifest high levels of distress with clinical relevance, in addition to anxiety, depression and anger. The emotional stress resulting from this stressful life-event, along with instrumental family support, may later be expressed in the grandparents’ physical and functional decline [22]. Furthermore, Findler [23] found that a higher level of stress is related to a perceived lower legitimacy of grandparents expressing how they feel. More recently, Lockton, Oxlad, and Due [24] concluded that grandparents’ emotional expression does not reflect their extent of psychological distress.

Hospitalizations represent a period of great disruption for the family system and in particular for grandparents, who feel the unpredictability and instability of their grandchildren’s illness resonate with their own vulnerability and finitude of life. Their main challenge is to witness and accept this suffering, along with the powerlessness of being unable to minimize it [18]. Nevertheless, grandparents acknowledge that they are not the protagonists in this context and tend to play their role in the background, aiming to maintain family stability and integrity [25].

From a systemic perspective, grandparents’ roles and care are complex and vary depending on family structure, needs and idiosyncrasies [26]. Regarding their tasks and responsibilities in the family, grandparents contribute with financial support, by taking care of their sick and healthy grandchildren, by providing emotional and spiritual support to their children, taking full or partial responsibility for household chores and making themselves available to stay with their grandchildren for hours or days, seeking to promote respite for their children [19].

Grandparents increase their efforts to live one day at a time and always try to do their best for their family, contributing to the circulation of communication among everyone and with health professionals [18]. This population has increased their use of remote communication platforms, particularly through video calling, to maintain emotional proximity with their children and grandchildren in the face of physical distance [26]. Kelada and colleagues [27] found that grandparents tended to report worse family functioning when they lived further away from the household of their sick grandchildren, reinforcing the importance of maintaining intrafamily communication.

During and after the experience of a potentially traumatic event, such as experiencing a child or youth’s complex chronic illness, and despite its painful and distressing impact, it is possible to observe some positively connoted psychological changes [28]. Grandparents have manifested post-traumatic growth through their perceived unconditional love, learning about health and illness, altruism, solidarity and spirituality [18]. This family subsystem also highlights pride regarding the perception of their skills and role as grandparents, greater affective proximity with their children, grandchildren and siblings and finding a new meaning in life [22].

In brief, it is noteworthy that research on grandparents in this field is mostly quantitative in nature, although there are several mixed methodology studies and qualitative studies based on interview scripts, conducted in person and by telephone, with genograms and direct observation in the context of hospitalization. In fact, qualitative methodologies have been considered the best means of exploring, specifying and explaining phenomena and processes that are characteristic of each context. The semi-structured interview is particularly interesting in its flexibility and invitation to reflection and psychotherapeutic relationships, within the scope of information collection in the context of psychological research and assessment [29].

### 1.3. Psychological Assessment

A comprehensive psychological assessment occurs in the affective, cognitive, structural, behavioral and communication domains, using various techniques—such as observation, interview, psychometric instruments and projective tests—and sources, according to a plan with a view to responding to previously established questions [30,31]. Additionally, this assessment includes the different members of the family system, along with the relational dynamics among them, the extended family and the contexts in which they act and influence each other [10,15].

Hence, the construction and development of scientifically supported and theoretically and methodologically anchored [29] assessment methodologies for grandparents of children/youths in the context of PPC [18] is sorely needed. It is also essential that the development of these methodologies allows grandparents to share their own perspective and deepen their narrative in a flexible manner, thus filling a gap in the scientific research.

This clinical tool proposed for assessing the experience of grandparents should be used for research and clinical purposes, contributing to a deeper understanding of the specific experiences of this population and promoting the research–intervention dialogue by facilitating its application by psychologists who work in this context for their psychological intervention. This comprehensive assessment tool enables a more oriented and personalized collection of information on the different dimensions of the psychological experience of this family subsystem, considering the heterogeneity of life-limiting and life-threatening complex chronic diseases.

Finally, it is essential to contribute to the expansion of systemic psychological intervention in pediatric palliative care so as to promote guidelines that foster the further development of grandparents’ wellbeing, coping, family resilience and post-traumatic growth in a holistic and dynamic approach.

### 1.4. Purpose of the Study

This study proposes a methodology for assessing the psychological experience of grandparents of children/youths with a life-limiting condition. Through its implementation, it seeks to contribute to an understanding of the psychological experience of the illness from their own perspective, which is essential in a population whose presence and role is under-explored in the literature and clinical practice.

Upon completion of the literature review, the objective was established to explore the following themes: representation of the illness; representation of the sick grandchild; changes in routine and life; family impact; grandparents’ contributions to the family system; social support and coping strategies; emotional impact; triple concern; needs identification; and post-traumatic growth.

This qualitative tool makes it possible to explore the specific challenges, features and coping strategies of grandparents’ psychological experience, resulting in the identification of specific needs and resources, as well as risks and protective factors, in an eco-systemic approach. Finally, it promotes the development of skills and boosts family resilience and post-traumatic growth among this population.

## 2. Materials and Methods

### 2.1. Design

This study uses a qualitative and exploratory design and was approved by the Comissão Especializada de Deontologia do Conselho Científico (Board of the Ethical and Deontological Committee) of the Faculty of Psychology, University of Lisbon.

### 2.2. Participants

The proposed assessment tool is intended for grandmothers and grandfathers of children/youths with a life-limiting or life-threatening disease [8]—diagnosed at least 12 months ago. The grandchild must be 18 years of age or younger. The grandparents must have an active role in the family nucleus but cannot be the primary caregivers of the grandchildren. It should be noted that participation is individual and the grandparents must be able to read, write or speak in English, unless the psychologist/researcher can do so for the participant.

### 2.3. Procedure

After identifying the singularities of the grandparents’ psychological experience in the context of PPC, anchored on an extensive and updated literature review, key dimensions deemed important to explore in a psychological assessment were defined. These dimensions were the starting point for the design of questions aimed at collecting targeted and comprehensive information.

The rationale behind the construction of the questions focused on formulating creative questions that would not induce stress or emotional activation, and would promote relief resulting from the opportunity for expression through a structured and contextualized narrative. The questions were organized in a sequential manner, based on an incomplete narrative involving the idea of sharing a testimony with other grandparents, since they represent the people who best understand their experience. This script was constructed on the basis of the Narrative Therapy strategies [32], where some guiding questions are asked, and the content of each participant’s answers reflects their dominant narrative about the way they experience the illness in its different stages [33]. Complementarily, a sociodemographic and clinical data sheet was prepared to characterize the grandparents and their personal and family circumstances. The structured clinical assessment tool of the grandparents’ psychological experience of the illness is thus composed of a sociodemographic and clinical data sheet and a semi-structured interview script based on an incomplete narrative. In a research context, this assessment tool should also be composed of an informed consent form to ensure compliance with ethical aspects.

### 2.4. Analysis

The responses resulting from this assessment tool, namely the script, should be understood using a reflexive thematic analysis of the narrative, through an inductive–deductive process [34,35].

## 3. Results

The script proposed within this methodology for assessing grandparents’ psychological experience of illness is presented in its entirety below.

### 3.1. Sociodemographic and Clinical Data Sheet

This sheet aims to collect initial information that characterizes the grandfather/grandmother and their family life, namely, through data regarding the grandparent him/herself, the grandchild with the life-limiting disease and the family [36]. The following information is collected as part of this sheet.

#### 3.1.1. Data Related to the Grandfather or Grandmother

Information regarding the following variables is requested: family relationship, age, gender, nationality, marital status, district of residence, education, occupation, employment status, past and present use of psychiatric/mental health services and physical health.

#### 3.1.2. Data on the Grandchild with Life-Limiting Conditions

Information is collected on age, gender, illness diagnosis, time elapsed since diagnosis, technological dependence, hospitalizations (number and last time), level of disability of the grandchild, institutional support, primary caregiver and secondary caregivers (if any) and knowledge regarding pediatric palliative care.

#### 3.1.3. Family Data

The aim of collecting this data is to gain additional knowledge about the family’s circumstances, namely, grandparent’s household (number of members, apart from the participant) and grandchildren’s household (number of members apart from the sick grandchild) with a characterization of all the members (according to gender, age, school attendance and/or professional occupation), as well the geographic distance between the two homes.

### 3.2. Semi-Structured Interview Script

This script is based on an incomplete narrative which involves sharing a testimony with other grandparents. The main purpose of using this strategy is to create an opportunity for emotional expression through the sharing of an intimate and unique experience. In addition, it aims to promote identification with family members in identical circumstances, therefore facilitating the perception of understanding and empathy, decreasing the activation of defense mechanisms and increasing collaboration and motivation [24].

The narrative presented in this script includes 15 interspersed questions which refer to the 10 dimensions under assessment. These dimensions correspond to the particularities of the illness-adaptation process among this population and were selected following the literature review, namely: representation of the illness; representation of the sick grandchild; changes in routine and life; family impact; grandparents’ contributions to the family system; social support and coping strategies; emotional impact; triple concern; needs identification; and post-traumatic growth. Each question, or set of questions, aims to operationalize each of the aforementioned domains. The script is presented in its entirely below.

*Imagine that right now there is a family with a child or teenager who has just received a diagnosis of a serious illness. There are grandparents in this family who are worried and who need to talk to other grandparents who are experiencing the same life situation. Throughout this platform, you will find some of the questions that these grandparents would like to ask you to help them make a more balanced adjustment to their grandchild’s illness. Your testimony is very important to them.*

*We recently learned that one of our grandchildren is ill and we are still thinking about all the information we have received. It would be very important for us to understand how you view your grandchild’s illness.*

***If your grandchild’s illness had an identity card, what three characteristics would you choose to define it?*** (Representation of the illness).

*Now imagine that we asked you to give a diploma to your sick grandchild.*

***What title would you give the diploma and what would you like to highlight about him/her?*** (Representation of the sick grandchild).

***How would you characterize your relationship with your grandchild?*** (Representation of the sick grandchild).

*At this time, we feel scared about the changes to our routine and in our life.*

***In your experience, what changes have you experienced since the diagnosis of the illness?*** (Changes in routine and life).

*In addition, we are concerned about how our family will adapt to this new situation, although we know it will have a major impact on all of us.*

***Thinking about how your family relates to each other, how they communicate and how they support each other, what do you consider to be the main changes that have occurred in your family?*** (Family impact).

*However, we are committed to trying to help our family, although we have not yet identified the best way of doing so.*

***What kind of things do you do for your family?*** (Grandparents’ contributions to the family system).

***If your family gave you a trophy for “best grandma(grandpa),” what three characteristics do you think they would highlight?*** (Grandparents’ contributions to the family system).

***What behaviors have you had that make you proud of your role as grandmother/grandfather?*** (Grandparents’ contributions to the family system).

***If you could do something different for your family that you have never done, what would it be?*** (Grandparents’ contributions to the family system).

*Thank you. Could you also help us understand how we can best get through this experience?*

***If you were to praise three people who have been very supportive and important throughout this period, who would they be and what would you say to them?*** (Social support and coping strategies)

***If you could give us three pieces of advice that have helped you cope better with your grandchild’s illness, what would they be?*** (Social support and coping strategies)

*Nevertheless, we believe that throughout this period you have thought and felt many different things. There may have been times when you felt sad and worried and other times when you managed to find some joy.*

***If what you feel were a puzzle with different sized pieces, what emotions would form the larger pieces?*** (Emotional impact).

*Several grandparents in the same situation report feeling invisible and prefer to silence their suffering, focusing only on the needs of their children and of both their sick and healthy grandchildren.*

***From your experience to what extent do you identify with the concerns of these family members?*** (Triple concern).

***Now we would like to ask you to think about what you have experienced and to share with us what challenges you think a grandparent in these circumstances might feel and that might not be understood by others?*** (Needs identification).

*Nevertheless, when we live with our grandchild’s illness for some time, we believe that we begin to discover some positive changes in ourselves, in our life, and in our family. Several people regard these changes as their personal growth.*

***What interesting discoveries have you made about yourself and your family?*** (Posttraumatic growth).

*Your interview has come to an end. Thank you very much for your participation in this study and for the testimony you have given to other grandparents who are now embarking on this path with their family, alongside the illness of a grandchild.*

## 4. Discussion

Through the application of this clinical tool, the researcher/psychologist can access the specific features of the grandparents’ psychological experience, which represent the psychological intervention themes proposed for this population. This will lead to an identification of concrete needs, as well as strategies, resources and competencies that may benefit from development or mobilization, in order to promote the family’s wellbeing and resilience [37]. It is particularly important because, in fact, more resilience skills, more social support and better family functioning are related to greater child wellbeing [38].

This assessment tool can be used in person or remotely (e.g., face-to-face interview, telephone call, online questionnaire or letter) and the participants’ privacy should be ensured in both contexts (e.g., through hospital, institutions, associations, at home or communication platforms). In addition, it can be used alone, in its entirety or combined with other psychological assessment instruments. The researcher/psychologist should guide the application of this clinical tool towards the dimensions that prove to be most useful and/or necessary in each family. The application will last approximately 45 min.

Indeed, it was designed so that it may be applied more than once to the same participant if it is properly phased in waves, providing an opportunity to explore any changes in the narrative over time, since the construction of an alternative narrative may be the result of a process of co-construction experienced within the scope of psychological intervention [33]. It should also be noted that the grandparents’ own application of the tool can translate into a therapeutic effect for them, as it represents an opportunity for emotional expression and the psychological organization of their feelings and thoughts [39].

Nevertheless, this assessment tool proposal has some limitations that need to be addressed. First, there is no universal model of the psychological experience of grandparents and the dimensions deemed more important and/or necessary to assess. This suggests that, according to specific socio-cultural, institutional and family circumstances, there may be other dimensions that could also be incorporated into the assessment. Second, there is an inherent limitation to any psychological assessment process, which is the probabilistic nature of its conclusions. Although it is important to consider these factors, this tool should be used in clinical practice with the construction of a positive therapeutic relationship.

## 5. Conclusions

In child and youth healthcare, the presence and tasks of grandparents have remained underexplored, both from the perspective of research and clinical intervention. In addition, a notable scarcity of empirically grounded theoretical and methodological frameworks specifically geared towards assessing the experience of grandparents has been observed. Consequently, there are minimal guidelines available to health professionals, and specifically to psychologists, to explore and identify the challenges among this population.

It is, therefore, essential to build and develop robust assessment processes within the scope of pediatric palliative care for specific populations, namely, the grandparents’ family subsystem. Its implementation also aims to organize the collection and streamline the integration of information on each specific grandparent in the context of his or her family, considering key dimensions for their in-depth understanding.

This study proposes a clinical tool to assess the psychological experience of grandparents of the life-limiting conditions of their grandchildren, based on a predominantly qualitative methodology. Its structure, organization and flexibility promote its application in different contexts, resulting from an attempt to respond to the clinical and research needs of professionals seeking to focus on the family members of children and young people with serious illnesses. The knowledge that may result from the implementation of this tool is fundamental for the identification of grandparents’ needs, challenges, motivations and resources.

The final goal is to promote a more adjusted and personalized definition of the objectives and strategies of psychological intervention, as well as to monitor the whole multidisciplinary team in health contexts, thus contributing to grandparents’ psychological wellbeing, resilience and post-traumatic growth, considering their personal, family and socio-cultural circumstances.

The authors consider that the proposed assessment tool may contribute to the scientific development of the psychological assessment of grandparents in the context of pediatric palliative care, and its application is recommended in studies in this field and in clinical practice with families in these circumstances. To this end, it is suggested that scientific studies be carried out on the implementation of this tool, allowing the development of focus groups with families and health professionals to evaluate the questions and a follow-up evaluation of the therapeutic benefits of its application and effectiveness.

## Data Availability

Data is contained within the article.
